# Fabrication of Periodontal Membrane From Nelumbo nucifera: A Novel Approach for Dental Applications

**DOI:** 10.7759/cureus.59848

**Published:** 2024-05-07

**Authors:** Ojastha BL, Suganya Panneer Selvam, Ramya Ramadoss, Sandhya Sundar, Pratibha Ramani, Bargavi P

**Affiliations:** 1 Oral Biology, Saveetha Dental College and Hospitals, Saveetha Institute of Medical and Technical Sciences, Saveetha University, Chennai, IND; 2 Oral Pathology and Microbiology, Saveetha Dental College and Hospitals, Saveetha Institute of Medical and Technical Sciences, Saveetha University, Chennai, IND

**Keywords:** regeneration, periodontitis, anti-inflammatory, antibacterial, periodontal ligament

## Abstract

Background

The periodontal membrane plays a crucial role in tooth support and maintenance. Natural materials with biocompatible and bioactive properties are of interest for periodontal membrane fabrication. *Nelumbo nucifera*, known for its therapeutic properties, presents a potential source for such materials.

Aim

This study aimed to fabricate a periodontal membrane from *N. nucifera* and evaluate its biocompatibility and potential for periodontal tissue regeneration.

Materials and methods

*N. nucifera* stems were collected dried, and aqueous extract was prepared. The extracted material was then processed into a membrane scaffold using a standardized fabrication method. The fabricated membrane was characterized by its physical and chemical properties. Biocompatibility was assessed using human periodontal ligament fibroblast (hPDLF) cells cultured on the membrane, followed by viability, proliferation, and anti-microbial assays.

Results

The fabricated *N. nucifera* membrane exhibited a porous structure with suitable mechanical properties for periodontal membrane application. The membrane supported the adhesion, viability, and proliferation of hPDLF cells in vitro.

Conclusion

The fabrication of a periodontal membrane from *N. nucifera* shows promise as a natural and biocompatible material for periodontal tissue regeneration. Further studies are warranted to explore its clinical potential in periodontal therapy.

## Introduction

*Nelumbo nucifera*, commonly referred to as lotus, is an aquatic perennial plant that falls within the *Nelumbonaceae* family. This plant serves as a valuable source of herbal medicine, exhibiting potent antipyretic, cooling, astringent, and demulcent properties. The seeds of the lotus plant are utilized in the preparation of various Ayurvedic remedies for conditions, such as tissue inflammation, cancer, diuretic effects, and skin diseases. Furthermore, these seeds are rich in compounds, particularly flavonoids, making them effective as an antidote for poisoning [1]. Previous studies have documented the capacity of lotus seeds to scavenge free radicals and protective effects against cytotoxicity and DNA damage generated by reactive nitrogen, sodium nitroprusside (SNP), peroxynitrite, and macrophage RAW 264.7 cell lines. [2]. Various studies have used finite element analysis (FEA) to develop constitutive models for the periodontal ligament (PDL) and understand its mechanical behavior. These models have considered the anisotropic, inhomogeneous, and non-linear elastic nature of the PDL [3,4].

Lotus seeds have been extensively studied for their antioxidant properties. Research has shown that different parts of the lotus plant, such as the seed epicarp, seed embryos, and rhizome knots, exhibit significant antioxidant activity due to their high phenolic content [5]. Lotus seed protein hydrolysate (LSPH) derived from lotus seeds has also demonstrated potent antioxidant abilities, with high scavenging activity against DPPH and H2O2 radicals [6]. Lotus root polysaccharide (LRP) and its carboxymethylated form have also been found to be natural antioxidants. The carboxymethylated form is better at getting rid of ferrous ions and hydroxyl radicals [7]. Furthermore, extracts from *Ziziphus lotus* L. seeds have exhibited antioxidant properties, with the methanol extract showing the highest DPPH radical scavenging activity [8]. These findings collectively highlight the free radical scavenging potential of lotus seeds and related plant parts.

Barrier membranes used in PDL regeneration require specific biocompatibility requirements, such as high biocompatibility, low cellular permeability, tight tissue adhesion, moderate mechanical strength, storage stability, and handleability. Current limitations of these membranes include weak biocompatibility of nonabsorbable and absorbable synthetic polymer membranes [9]. Furthermore, it should be noted that natural collagen membranes demonstrating rapid degradation rates possess restricted rigidity and may not effectively sustain barrier functionality [10,11]. To improve biocompatibility, possible solutions include using electrospinning techniques, nanofiber scaffolds, or developing functional gradient membranes [12]. Among them, clinicians currently use membranes made of poly-lactic acid (PLA) and poly-glycolic acid (PGA) that exhibit a diverse range of tensile strength, ranging from 40 to 140 Mpa. However, these membranes generate harmful oxidative species when they are broken down by polymorphonuclear leukocytes, and the material is directly proportional to the inflammatory response, thus leading to the failure of the treatment [13]. Hence, the aim of this research is to fabricate a PDL membrane/barrier membrane naturally from the stem of *N. nucifera*.

## Materials and methods

Preparation of the PDL membrane from the stem extract

**Figure 1:**
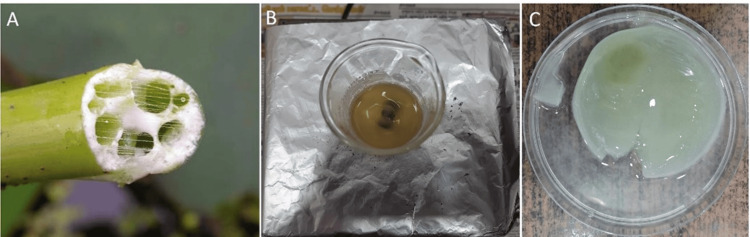
Preparation of membrane from the stem of Nelumbo nucifera (A: stems were segregated, B: the extract preparation, C: membrane prepared from the extract)

*N. nucifera* is collected from local flower shops. Stems were removed, dried and crushed into powder, boiled in water, and filtered the extract, and the filtrate obtained is the aqueous extract (Figure 1A, 1B, 1C). Five grams of the extract was mixed with 20 ml of sodium alginate and placed in a stirrer for five to 10 minutes. The polymer mixture was poured into the petri dish and kept overnight at -20 ºC after the complete mixture of seed extract in the alginate polymer. The membrane formed instantly was stored in the freezer, and 6% calcium chloride (a cross-linking solution that maintains the same solid state of the membrane formed out from the freezer) solution was added above the membrane and kept aside. Thus, the thin membrane was fabricated. The shelf life of a PDL membrane fabricated from plants can vary depending on the specific material and how it is processed and stored. If the membrane is properly processed, packaged, and stored, it can have a shelf life of around two to three years.

Membrane morphology

Morphological analysis was done using a scanning electron microscope (JEOL-JSM-IT800 electron microscope, JEOL, Ltd., Japan) at 1 Kv. The samples were dried up before SEM analysis with the help of a critical point dryer (Leica EM CPD300, Leica Microsystems GmbH, Germany).

Hemocompatibility assessment

The biocompatibility test was assessed by mixing 50 µl with 950 µl of double distilled water for the positive control and mixing 50 µl with 950 µl of phosphate-buffered saline (PBS) for the negative control. The solution was exposed to the membranes, and the rate of hemolysis was assessed using the positive and negative controls.

Antimicrobial activity

The antibacterial activity of the extract was evaluated against strains of *Streptococcus mutans*, *Enterococcus faecalis*, and *Staphylococcus aureus* using Muller-Hinton agar in order to ascertain the zone of inhibition. The agar medium was prepared, sterilized, and permitted to solidify for a duration of 16 minutes at a temperature of 121 °C. The test organisms were collected by swabbing them following the incision of the wells using a 9 mm sterile polystyrene tip. The sample was loaded with various concentrations (5, 10, 15, and 20 microliters) and the area of inhibition was assessed after incubating the plates at 37°C for 24 hours. The activity of Candida albicans is assessed through the application of the Agar Well Diffusion Assay, employing Rose Bengal Agar as the medium. Prior to the addition of varying volumes of the extract, specifically 5, 10, 15, and 20 microliters, the wells were subjected to a sterile medium that had been effectively swabbed with the test pathogen. The plates were raised at a temperature of 37 °C for a duration of 40-72 hours. Following the designated incubation period, the zone of inhibition was assessed.

Confocal microscope

The hPDLF cells were washed with PBS and fixed with formaldehyde. The cells were labeled with fluorescent dyes like acridine orange and proprium iodide targeting their attachments over the synthesized membrane. The labeled cells were imaged using a confocal laser scanning microscope.

## Results

Morphological analysis of the membrane

**Figure 2:**
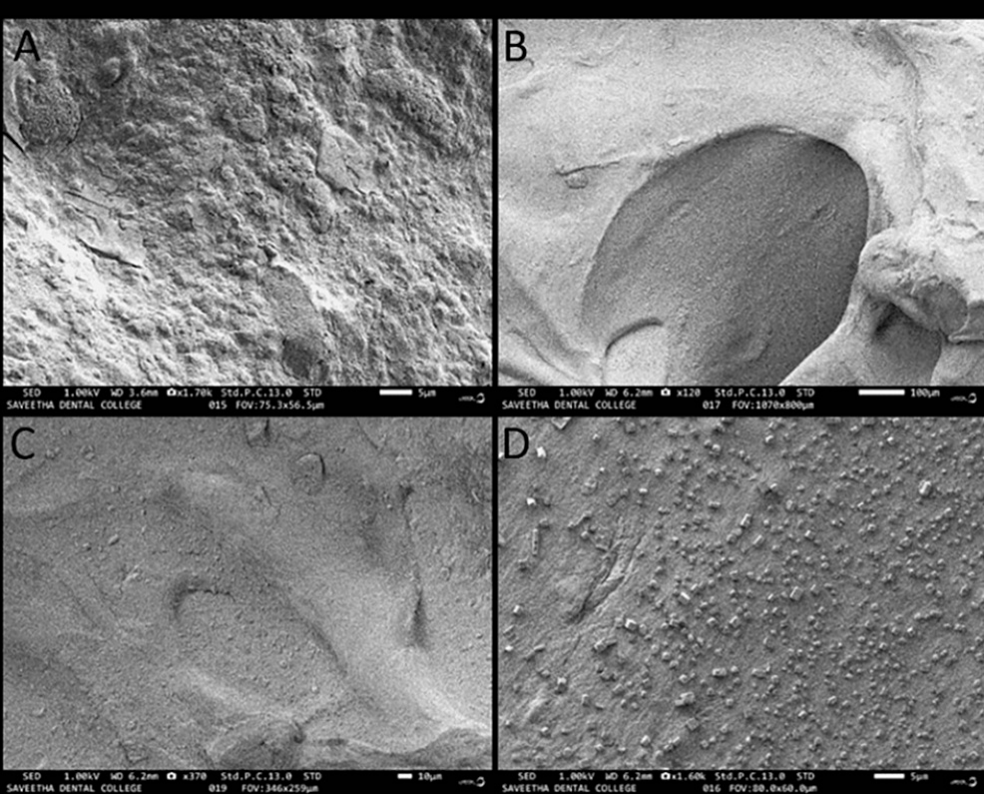
A and B show the smooth alginate membrane. C and D depict the membrane fabricated from Nelumbo nucifera displayed surface roughness.

The alginate membrane exhibited a smooth surface characterised by few imperfections and irregularities, as depicted in Figure 2. The *N. nucifera* membrane exhibited surface roughness, resulting in an increased surface area. The findings indicate that the extended endurance of the material can be attributed to its robust crystalline structure. The incorporation of the stem extract has the potential to enhance the surface roughness of the membrane, hence facilitating the adhesion of cellular components to the hydrogel membrane.

Hemocompatibility of the membrane

**Figure 3:**
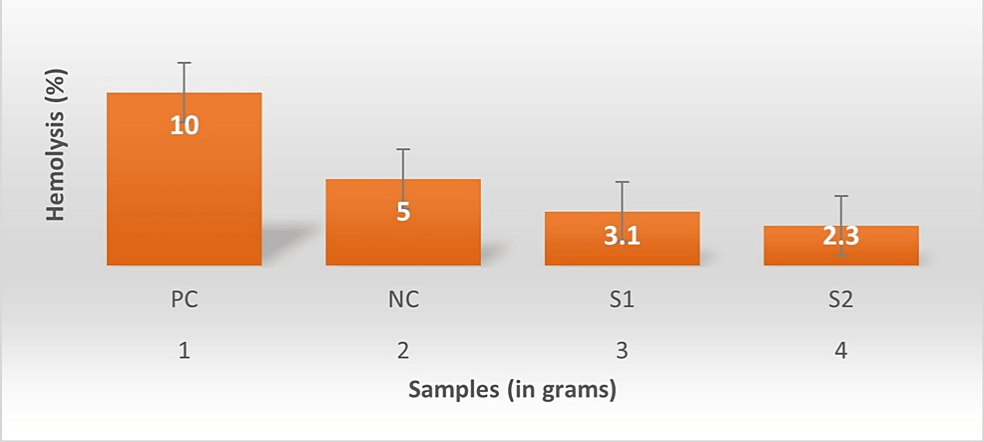
Hemolysis of the fabricated membrane with improved biocompatibility with increasing concentration PC: positive control, NC: negative control, S1: sample 1 (5 grams), S2: sample 2 (10 grams)

The membrane's biocompatibility was evaluated using both positive and negative controls. Both solutions were applied to the membrane, and the rate of lysis was quantified using a UV spectrophotometer. The PDL membrane derived from *N. nucifera* exhibited a hemolysis rate of less than 5% (Figure 3).

Antimicrobial assessment of the membrane

**Figure 4:**
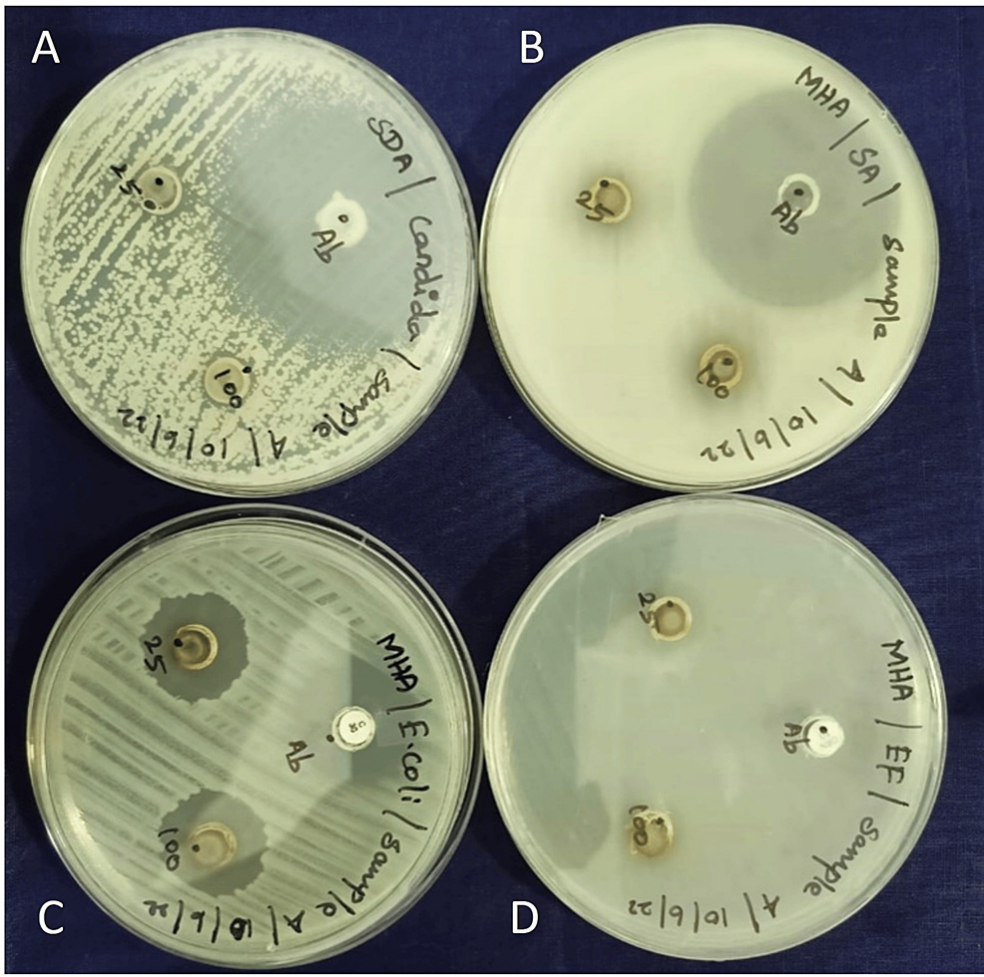
Antimicrobial activity of the membrane fabricated from Nelumbo nucifera against Candida albicans (A), Staphylococcus aureus (B), Escherichia coli (C), Enterococcus faecalis (D)

Although the membrane has good biocompatibility, the antibacterial activity against *S. aureus *and *E. coli* was very mild. The antimicrobial activity of the membrane against *E. faecalis* and *Candida albicans* were negative (Figure 4).

Confocal imaging of the cells in the membrane

**Figure 5:**
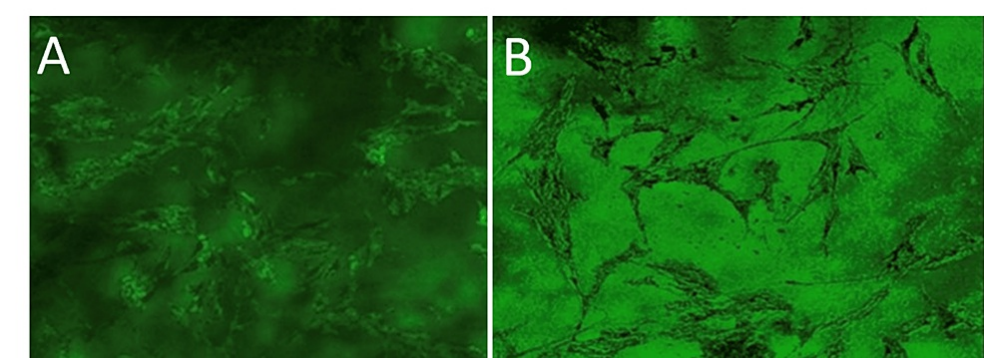
A and B show the attachment of human periodontal ligament fibroblast cells to the membrane with spindle-like processes over the fabricated membrane under a confocal microscope

The adhesion, proliferation, and focal adhesion formation of the cultured human periodontal ligament fibroblast cells (hPDLF) cells were observed by confocal laser microscopy. The encapsulated hPDLF cells were homogenously distributed inside the matrix of the polymer membrane. After incubation for 24 hours, the cells were well attached to the lotus stem extract blended alginate membrane. The figure shows the uniform cytoskeleton of the hPDLF cell network interconnecting the polymer membrane. The phenomenon of cell spreading is evident, as the cells that are connected exhibit spindle-like processes (Figure 5).

## Discussion

Plant-based PDL membranes are gaining significant attention in research due to their high biocompatibility [14,15]. These membranes, derived from plant sources like olive leaves and *Lythri herba*, offer non-toxic, eco-friendly, and renewable alternatives for wound dressings and cell culture platforms [16,17]. The use of plant-derived compounds in biomaterials enhances biocompatibility and accelerates tissue repair processes, making them ideal for applications in regenerative medicine [18]. In addition, decellularized plant-based scaffolds provide a natural and tunable substrate for 3D cell culture, promoting cell communication and differentiation. The versatility and effectiveness of plant-based PDL membranes highlight their potential as innovative solutions in biomedicine, offering promising avenues for further research and development. Our research is the first of its kind to fabricate a periodontal membrane from the stem of *N. nucifera*.

A previous study evaluated the antibacterial activity of the scaffold with *Ziziphus jujuba* and found it to be microbicidal against *S. aureus* and *E. coli* [19]. A recent study in fabricating the GTR membrane using the mucilage of Chia seeds and lignin-mediated ZnO nanoparticles showed remarkable antibacterial properties against *S.aureus *and *E. coli* with good degradation properties [20]. These findings are consistent with our study and suggest that this new approach could be a promising solution for combating bacterial infections.

In recent times, considerable research has been conducted on the composite scaffold, a biomaterial structure composed of two or more different materials, usually in the form of a matrix or framework, which are combined to create a scaffold with enhanced properties for tissue engineering and regenerative medicine applications. These scaffolds have proven to be highly effective in promoting cell adhesion, proliferation, and differentiation, as well as facilitating tissue regeneration in a variety of applications. One such composite scaffold synthesized from bacterial cellulose, chitosan, and hydroxyapatite was studied for the release of pomegranate peel extract. The study found that the release of the extract was dose-dependent and had a microbicidal effect with good wound-healing abilities [21,22]. However, the precise control of concentration and its cost in fabrication, and interfacial bonding is still a problem. Hence, researchers need to consider the correlation between scaffold parameters and cell fate, as well as the effects of biochemical characteristics, structure architecture, biodegradability, and mechanical behavior of scaffold materials, in tissue engineering [23].

Hydroxyapatite was infused with the alginate membrane (HAp-Alg) and was found to have good biocompatibility with the alginate and pH-responsive degradability [24]. Another novel innovation for periodontal regeneration includes bacterial/plant-derived extracellular vesicles (BEVs/PEVs) has been shown to play a role in periodontal homeostasis and regeneration [25,26]. These vesicles are secreted by bacteria and plants and contain biomolecules that mediate communication between cells. Studies have demonstrated that BEVs and PEVs have beneficial effects on periodontal regeneration, making them a potential alternative strategy for cell-based periodontal regeneration [27].

Therefore, there is a need for further in-depth research to understand the complex interactions between scaffold materials and cells. The development of advanced composite scaffolds that can mimic the natural extracellular matrix and provide an optimal microenvironment for cell growth and differentiation is crucial. The integration of multiple materials with different properties to create a composite scaffold with enhanced physical, biological, and mechanical properties is also an important area of focus.

Limitations of the study

The periodontal membrane fabricated from *N. nucifera* has demonstrated exceptional biocompatibility. However, the present study was limited to the evaluation of biocompatibility and potential for regeneration in vitro, which may not fully represent the complex in vivo environment of periodontal tissues. The biocompatibility was evaluated using hPDLF cells only. The interaction of the membrane with other cell types involved in periodontal regeneration, such as osteoblasts or immune cells, will be considered in the future. While the study suggests promise for clinical use, further research, including animal studies and clinical trials, is needed to validate its safety and efficacy in humans.

## Conclusions

The fabrication of a periodontal membrane from *N. nucifera* demonstrates promising potential as a natural and biocompatible material for periodontal tissue regeneration. The membrane exhibited suitable physical and mechanical properties and supported the adhesion, viability, and proliferation of hPDLF cells in vitro. These findings suggest that *N. nucifera* could be a viable candidate for use in periodontal therapy. However, further studies, including in vivo investigations and clinical trials, are warranted to validate its efficacy and safety for clinical application.
